# Analysis of Mineral Waters

**Published:** 1853-04

**Authors:** 


*Analysis of Mineral Waters.* (From the Southern Journal of the Medical and Physical Sciences.)

1. *Nashville Sulphur Spring*—Prof. Bowen:
   Sulphuretted Hydrogen,
   Carbonic Acid,
   Hydrochloric Acid,
   Sulphuric Acid,
   Magnesia as a Sulphate,
   Soda as a Hydrochlorate.

2. *Sam's Creek Spring, Davidson Co.*—Prof. Troost:
   Twenty Fluid Ounces.
   Sulphuretted Hydrogen,
   Carbonic Acid,
   Sulphate of Lime,
   Hydrochlorate of Soda.

3. *Tyree's Springs, Davidson Co.*—Prof. Troost:
   Sulphuretted Hydrogen,
   Carbonic Acid,
   Sulphate of Lime,
   Sulphate of Magnesia,
   Carbonate of Lime,
   Hydrochlorate of Soda.

4. *Shelby Chalybeate Spring, Nashville* — Richard O. Currey:
   Temperature of Spring 61°, while air was 90°.
   Carbonic Acid, free,
   Carbonate of Iron,
   Carbonate of Magnesia,
   Carbonate of Lime,
   Chloride of Sodium,
   Sulphate of Magnesia.

5. *Bayley's Spring, Florence, Ala.*—Richard O. Currey:
   Carbonic Acid, 324 cubic inches in a gal.,
   Carbonate of Magnesia,
   Carbonate of Soda,
   Carbonate of Iron,
   Carbonate of Potash,*

---

* According to Prof. Tuomey.

Iodine,

Chloride of Sodium.

[The analysis of this spring was made in June, 1852, in reference to the quality, not the quantity, of its ingredients. There were distinct indications of iodine present, to the amount at least of three grains to the gallon of water. It probably exists in combination with the potash as an hydriodate. These springs are noted for their efficacy in scrofulous and dropsical diseases.]

6. *Congress Spring at Saratoga*—Steel :

| | | |
|---|---|---|
| Carbonic Acid, | cubic in. | 311 in a gallon. |
| Atmospheric Air, | | 7 |
| Chloride of Sodium, | grs. | 385 |
| Iodide of Sodium, | | 3.5 |
| Bicarbonate of Soda | | 8.98 |
| Bicarbonate of Magnesia, | | 95.78 |
| Carbonate of Lime, | | 98.09 |
| Carbonate of iron, | | 5.07 |
| Silica, | | 1.5 |
| Bromide of Potassium, a trace. | | |

7. *White Sulphur Spring, Va.*—Prof. W. B. Rogers :

| | | |
|---|---|---|
| Sulphuretted Hydrogen, | cubic in. | 2.5 in a wine gallon. |
| Carbonic Acid, | | 2 |
| Oxygen, | | 1.4 |
| Nitrogen, | | 3.5 |
| | Total, | 9.4 |

Solid contents in a pint.

| | | |
|---|---|---|
| Sulphate of Magnesia, | grs. | 5.5 |
| Sulphate of Lime, | | 7.7 |
| Carbonate of Lime, | | 1.1 |
| Chloride of Calcium, | | 0.2 |
| Chloride of Sodium, | | 0.1 |
| Oxide of Iron, a trace. | | |
| Loss, | | 0.4 |
| | | 15.0 |

8. *Iodine Spring, at Saratoga*—Prof. Emmons:

| | | |
|---|---|---|
| Carbonic Acid, | cubic in. 336 in a gallon. | |
| Atmospheric Air, | 4 | |
| Chloride of Sodium, | grs. 187 in a gallon. | |
| Carbonate of Magnesia, | 75 | |
| Carbonate of Lime, | 26 | |
| Carbonate of Soda, | 2 | |
| Carbonate of Iron, | 1 | |
| Iodine, | 3.5 | |
| | 294.5 | |

